# Supplementary material for: Directed acyclic graphs and causal thinking in clinical risk prediction modeling
Source: BMC Med Res Methodol. 2020 Jul 2;20:179. doi: 10.1186/s12874-020-01058-z (PMC7331263; doi:10.1186/s12874-020-01058-z)
Supplement: Supplementary file 1 — Additional file 1. [file 12874_2020_1058_MOESM1_ESM.pdf]

Supplementary file - R code

Directed Acyclic Graphs and causal thinking in clinical risk prediction modeling

Marco Piccininni, Stefan Konigorski, Jessica L Rohmann, Tobias Kurth

Overview

This is the R code that was used in order to conduct the simulation and generate the results described in the subsection "Predictor selection and the Markov Blanket" of the Results in the main manuscript. For illustrative purposes we present the code for only one DAG. In order to obtain results for 100,000 randomly generated DAGs, the foreach function in the doParallel package was used, parallelizing over 100 cores. It was run on R 3.6.3 on Ubuntu 18.04.

Load packages

```
library(dagitty)
library(glmnet)
library(randomForest)
```

Define the ICI function

This function is used to compute the Integrated Calibration Index (ICI), which we use as a metric to compare methods.

Reference: Austin & Steyerberg 2019, https://doi.org/10.1002/sim.8281

```
ICI <- function(outcome, prediction, main, plot = FALSE) {
  P.calibrate <- predict(loess(outcome ~ prediction))
  if (plot == TRUE) {
    plot(outcome ~ prediction, main = main)
    lines(prediction[order(prediction)], P.calibrate[order(prediction)], col = "red")
    abline(0, 1)
  }
  (ICI <- mean(abs(P.calibrate - prediction)))
}
```

Set parameters

```
k_cv <- 10 # number of cross-validation sets
ndatasim <- 10000 # sample size
n_nodes <- 25 # number of nodes in random DAG
probarrow <- 0.1 # probability that there is an arrow between two nodes
outcome_prob <- 0.2
param_outcome_1 <- -1
param_outcome_2 <- 1
param_pred_1 <- -2
param_pred_2 <- 2
plot_graphs <- FALSE # parameter to visualize plots
```

Define dataset to store the results

```
report <- data.frame(
  ICI_logistic_all = NA_real_,
  ncov_logistic_all = NA_integer_,
  ICI_logistic_dag = NA_real_,
  ncov_logistic_dag = NA_integer_,
  ICI_logistic_par = NA_real_,
  ncov_logistic_par = NA_integer_,
  ICI_logistic_mb = NA_real_,
  ncov_logistic_mb = NA_integer_,
  ICI_lasso = NA_real_,
  ICI_ridge = NA_real_,
  ICI_elastnet = NA_real_,
  ICI_rf = NA_real_,
  lasso_sel_mb = NA_real_,
  elastnet_sel_mb = NA_real_
)
```

Analysis

Below, we show the annotated simplified code for a single DAG.

Generate DAG

```
dag <- randomDAG(n_nodes, probarrow)

# find all the nodes
allnodes <- paste("x", 1:n_nodes, sep = "")

# choose one outcome to be predicted
outcome <- sample(allnodes, 1)
```

```r
# track all the nodes that have a path to the outcome
dagnodes <- c()
for (l in which(allnodes != outcome)) {
  connected <- length(paths(dag, from = outcome, to = allnodes[l], limit = 1)$paths)
  if (connected == 1) {
    dagnodes <- c(dagnodes, allnodes[l])
  }
}
```

Generate data for the variables in the DAG

```r
datadag <- data.frame(matrix(NA, ncol = length(allnodes), nrow = ndatasim))
names(datadag) <- allnodes
```

Generate exogenous variables

Generate all exogenous variables as N(0,1) (or binom(1,outcome_prob) if the exogenous variable is the outcome).

```r
datadag[, exogenousVariables(dag)] <- rnorm(ndatasim * length(exogenousVariables(dag)))
if (outcome %in% exogenousVariables(dag)) {
  datadag[, outcome] <- rbinom(ndatasim, 1, outcome_prob)
}
```

Generate endogenous variables

Create all other variables (including the outcome) as descendants of the respective parents.

```r
for (i in 1:n_nodes) {
  if (!(allnodes[i] %in% exogenousVariables(dag))) {
    covariates <- parents(dag, allnodes[i])
    if (allnodes[i] == outcome) {
      coeffs_out <- runif(length(covariates) + 1, param_outcome_1, param_outcome_2)
      pred <- as.matrix(cbind(1, datadag[, covariates, drop = FALSE])) %*% coeffs_out
      datadag[, allnodes[i]] <- rbinom(ndatasim, 1, prob = exp(pred) / (1 + exp(pred)))
    } else {
      coeffs <- runif(length(covariates) + 1, param_pred_1, param_pred_2)
      datadag[, allnodes[i]] <-
        as.matrix(cbind(1,datadag[,covariates,drop = FALSE])) %*% coeffs + rnorm(ndatasim)
    }
  }
}
```

Generate help dataset to store cross-validation results

```r
report_help_cv <- data.frame(
  ICI_logistic_all = rep(NA_real_, k_cv),
```

```
  ICI_logistic_dag = rep(NA_real_, k_cv),
  ICI_logistic_par = rep(NA_real_, k_cv),
  ICI_logistic_mb = rep(NA_real_, k_cv),
  ICI_lasso = rep(NA_real_, k_cv),
  ICI_ridge = rep(NA_real_, k_cv),
  ICI_elastnet = rep(NA_real_, k_cv),
  ICI_rf = rep(NA_real_, k_cv),
  lasso_sel_mb = rep(NA_real_, k_cv),
  elastnet_sel_mb = rep(NA_real_, k_cv),
  lasso_coef_char = rep(NA_character_, k_cv),
  elastnet_coef_char = rep(NA_character_, k_cv),
  stringsAsFactors = FALSE
)
```

Cross-validation

Fit models in k-fold cross-validation - always train model (i.e. obtain regression coefficients) using training data, then evaluate (apply model, get predictions, and assess performance) using test data.

```
for (k in 1:k_cv) {
  # preparation
  idx_test <- (k - 1) * (ndatasim / k_cv) + 1:(ndatasim / k_cv)
  idx_train <- which(!1:ndatasim %in% idx_test)
  train_data <- datadag[idx_train, ]
  test_data <- datadag[idx_test, ]
  train_data_predictors <- as.matrix(dplyr::select(train_data, -outcome))
  test_data_predictors <- as.matrix(dplyr::select(test_data, -outcome))

  # lasso with all variables
  # first identify best lambda in 10-fold cross-validation using the cv.glmnet function
  # then fit model to predict
  lasso_cv_res <- glmnet::cv.glmnet(x = train_data_predictors,
                                    y = as.factor(train_data[, outcome]),
                                    alpha = 1, family = "binomial")
  lasso_res <- glmnet::glmnet(x = train_data_predictors,
                              y = as.factor(train_data[, outcome]),
                              alpha = 1, family = "binomial",
                              lambda = lasso_cv_res$lambda.min)
  lasso_pred <- predict(lasso_res, s = lasso_cv_res$lambda.min,
                        newx = test_data_predictors, type = "response")
  report_help_cv$ICI_lasso[k] <- ICI(outcome = test_data[, outcome],
                                     prediction = lasso_pred, main = "lasso",
                                     plot = plot_graphs)
  lasso_coef <- data.frame(coef.name = dimnames(coef(lasso_res))[[1]],
                           coef.value = matrix(coef(lasso_res)))

  # ridge regression with all variables
  ridge_cv_res <- glmnet::cv.glmnet(x = train_data_predictors,
                                    y = as.factor(train_data[, outcome]),
                                    alpha = 0, family = "binomial")
  ridge_res <- glmnet::glmnet(x = train_data_predictors,
                              y = as.factor(train_data[, outcome]),
```

```r
                              alpha = 0, family = "binomial",
                              lambda = ridge_cv_res$lambda.min)
ridge_pred <- predict(ridge_res, s = ridge_cv_res$lambda.min,
                      newx = test_data_predictors, type = "response")
report_help_cv$ICI_ridge[k] <- ICI(outcome = test_data[, outcome],
                                   prediction = ridge_pred, main = "ridge",
                                   plot = plot_graphs)

# elastic net regression with all variables
elast_cv_res <- glmnet::cv.glmnet(x = train_data_predictors,
                                  y = as.factor(train_data[, outcome]),
                                  alpha = 0.5, family = "binomial")
elast_res <- glmnet::glmnet(x = train_data_predictors,
                            y = as.factor(train_data[, outcome]),
                            alpha = 0.5, family = "binomial",
                            lambda = elast_cv_res$lambda.min)
elastnet_pred <- predict(elast_res, s = elast_cv_res$lambda.min,
                         newx = test_data_predictors, type = "response")
report_help_cv$ICI_elastnet[k] <- ICI(outcome = test_data[, outcome],
                                      prediction = elastnet_pred,
                                      main = "elastic net", plot = plot_graphs)
elastnet_coef <- data.frame(coef.name = dimnames(coef(elast_res))[[1]],
                            coef.value = matrix(coef(elast_res)))

# random forest with all variables
rf_res <- randomForest(x = train_data_predictors,
                       y = as.factor(train_data[, outcome]),
                       xtest = test_data_predictors,
                       ytest = as.factor(test_data[, outcome]), ntree = 1000)
rf_pred <- rf_res$test$votes[, 2]
report_help_cv$ICI_rf[k] <- ICI(outcome = test_data[, outcome],
                                prediction = rf_pred,
                                main = "random forests", plot = plot_graphs)

# logistic regression with all variables
if (k == 1) {report$ncov_logistic_all <- length(allnodes[allnodes != outcome])}
formula_all <- as.formula(paste(outcome, paste(allnodes[allnodes != outcome],
                                               collapse = "+"), sep = "~"))
logistic_all <- glm(formula_all, train_data, family = binomial(link = "logit"))
logistic_all_pred <- predict(logistic_all, type = "response", newdata = test_data)
report_help_cv$ICI_logistic_all[k] <- ICI(outcome = test_data[, outcome],
                                          prediction = logistic_all_pred,
                                          main = "log reg, all variables",
                                          plot = plot_graphs)

# logistic regression with only parents
if (k == 1) {report$ncov_logistic_par <- length(parents(dag, outcome))}
if (!length(parents(dag, outcome)) == 0) {
  covar_par <- paste(parents(dag, outcome), collapse = "+")
} else {covar_par <- 1}
formula_par <- as.formula(paste(outcome, covar_par, sep = "~"))
logistic_par <- glm(formula_par, train_data, family = binomial(link = "logit"))
logistic_par_pred <- predict(logistic_par, type = "response", newdata = test_data)
```

```
  report_help_cv$ICI_logistic_par[k] <- ICI(outcome = test_data[, outcome],
                                            prediction = logistic_par_pred,
                                            main = "log reg, parents",
                                            plot = plot_graphs)

  # preparation for further logistic regression models
  if (k == 1) {report$ncov_logistic_dag <- length(dagnodes)}
  if (k == 1) {
    mb <- markovBlanket(dag, outcome)
    report$ncov_logistic_mb <- length(mb)
  }
  if (!length(dagnodes) == 0) {
    covar_dag <- paste(dagnodes, collapse = "+")
    covar_mb <- paste(mb, collapse = "+")
  } else {
    covar_dag <- covar_mb <- 1
  }

  # logistic regression with all variables in the DAG with a path to the outcome
  formula_dag <- as.formula(paste(outcome, covar_dag, sep = "~"))
  logistic_dag <- glm(formula_dag, train_data, family = binomial(link = "logit"))
  logistic_dag_pred <- predict(logistic_dag, type = "response", newdata = test_data)
  report_help_cv$ICI_logistic_dag[k] <- ICI(outcome = test_data[, outcome],
                                            prediction = logistic_dag_pred,
                                            main = "log reg, var with path",
                                            plot = plot_graphs)

  # logistic regression with all the variables in the Markov Blanket
  formula_mb <- as.formula(paste(outcome, covar_mb, sep = "~"))
  logistic_mb <- glm(formula_mb, train_data, family = binomial(link = "logit"))
  logistic_mb_pred <- predict(logistic_mb, type = "response", newdata = test_data)
  report_help_cv$ICI_logistic_mb[k] <- ICI(outcome = test_data[, outcome],
                                           prediction = logistic_mb_pred,
                                           main = "log reg, Markov blanket",
                                           plot = plot_graphs)

  # check coefficients
  report_help_cv$lasso_sel_mb[k] <-
    identical(
      as.character(lasso_coef[abs(lasso_coef$coef.value) > 10^-10 &
                              lasso_coef$coef.name != "(Intercept)", "coef.name"]), mb)
  report_help_cv$lasso_coef_char[k] <- paste(lasso_coef[, 2], collapse = ", ")
  report_help_cv$elastnet_sel_mb[k] <-
    identical(
      as.character(elastnet_coef[abs(elastnet_coef$coef.value) > 10^-10 &
                           elastnet_coef$coef.name != "(Intercept)", "coef.name"]), mb)
  report_help_cv$elastnet_coef_char[k] <- paste(elastnet_coef[, 2], collapse = ", ")
}
```

Average these values and store in overall results dataset

```
report$ICI_logistic_all <- mean(report_help_cv$ICI_logistic_all)
report$ICI_logistic_dag <- mean(report_help_cv$ICI_logistic_dag)
report$ICI_logistic_par <- mean(report_help_cv$ICI_logistic_par)
report$ICI_logistic_mb <- mean(report_help_cv$ICI_logistic_mb)
report$ICI_lasso <- mean(report_help_cv$ICI_lasso)
report$ICI_ridge <- mean(report_help_cv$ICI_ridge)
report$ICI_elastnet <- mean(report_help_cv$ICI_elastnet)
report$ICI_rf <- mean(report_help_cv$ICI_rf)
report$lasso_sel_mb <- mean(report_help_cv$lasso_sel_mb)
report$elastnet_sel_mb <- mean(report_help_cv$elastnet_sel_mb)
```
